# Partial loss-of-function of *NAL1* alters canopy photosynthesis by changing the contribution of upper and lower canopy leaves in rice

**DOI:** 10.1038/s41598-017-15886-5

**Published:** 2017-11-21

**Authors:** Naoki Hirotsu, Kazuhiro Ujiie, Ishara Perera, Ayano Iri, Takayuki Kashiwagi, Ken Ishimaru

**Affiliations:** 1 Graduate School of Life Sciences, Toyo University, 1-1-1 Izumino, Itakura, Oura, Gunma 374-0193 Japan; 2 Institute of Crop Science, National Agriculture and Food Research Organization, Kannondai 2-1-2, Tsukuba, Ibaraki 305-8518 Japan

## Abstract

Little is known about the genetic basis of leaf and canopy photosynthesis. Here we aimed to detect novel quantitative trait loci (QTL) controlling photosynthesis by increasing leaf nitrogen content (LNC) per leaf area and analysed its effect on leaf and canopy photosynthesis. To identify QTL that increase photosynthetic rate in leaves, we screened chromosome segment substitution lines (CSSLs) of *Oryza sativa* ssp. *japonica* cultivar Koshihikari and *O. sativa* ssp. *indica* cultivar Nona Bokra using LNC per leaf area as the phenotype indicator. Locus *leaf nitrogen content* on chromosome four (*qLNC4*) is associated with increased LNC and photosynthetic rate per leaf area. Moreover, a non-synonymous amino acid substitution was identified in the *NARROW LEAF 1* (*NAL1*) gene located in the *qLNC4* region. This *NAL1* allele increases LNC and photosynthetic rate per leaf area in flag leaves but does not increase whole-leaf photosynthesis. This *NAL1* allele also increases light capture and whole-leaf nitrogen content of the lower leaves and is associated with slower senescence in flag leaves. These results suggest that this *NAL1* allele does not increase whole-leaf photosynthesis but plays a role in regulating spatial and temporal trade-offs among traits at the whole-plant level.

## Introduction

Improving photosynthesis is important to increase biomass and crop yield in plant breeding. In general, the uppermost, fully expanded leaf displays the maximum rate of photosynthesis in a plant, a target for increasing yield potential through increasing photosynthetic rate per leaf area. The maximum photosynthetic rate per leaf area (Pn; μmol CO_2_ m^−2^ s^−1^) is correlated with leaf nitrogen content per leaf area (LNC; g m^−2^) in diverse rice (*Oryza sativa*) genotypes^1^ and C_3_ crops^2^. Although ribulose-1,5-bisphosphate carboxylase/oxygenase (Rubisco) is the primary CO_2_-fixation enzyme and the most abundant protein in leaves^3,4^, the Rubisco to LNC ratio is known to be constant in rice^5,6^. In addition, nitrogen (N) content strongly affects grain yield and plants’ biomass by limiting leaf photosynthetic ability^2,7^. Thus, controlling LNC is important for the regulation of photosynthesis and yield potential.

Understanding the genetic basis of LNC is indispensable for genetic improvement of photosynthesis. Quantitative trait loci (QTL) analysis is an efficient approach to identify loci or genes responsible for quantitative traits^8–10^. Ishimaru *et al*.^11^ reported rice varietal differences in QTL controlling LNC using backcross inbred lines (BILs) between Nipponbare and Kasalath cultivars. In the same mapping population, QTL for soluble protein content were associated with N-metabolism enzymes and yield-related traits^12^. Although the mechanism by which QTL control LNC is known, how these QTL affect leaf photosynthesis has not been elucidated so far.

The ability of a plant to produce carbohydrates is determined by canopy photosynthesis, i.e. the integration of leaf photosynthesis among the several layers of leaves^13^. In rice, the uppermost leaf during grain filling is the flag leaf, and, in conjunction with the lower leaf, contributes to carbohydrate accumulation in grains^14,15^. Although leaf area also determines the ability for leaf photosynthesis, excessively large leaves may cause shading and contribute to reduce overall canopy photosynthesis^16^. Some rice breeding programs set an erect leaf orientation as the ideotype of the morphological trait, as it allows light to penetrate through the canopy and reach the lower leaves^15^. Indeed, *Osdwarf4* mutants, in which erect leaves are obtained through functional redundancy in the brassinosteroid biosynthesis pathway, show increased biomass under dense planting conditions^17^. Therefore, to improve whole-plant photosynthesis, it is important to increase both the Pn in uppermost leaves and canopy photosynthesis. However, the genetic basis of canopy photosynthesis is still unknown.

Few studies have investigated QTL that increase maximum rates of leaf photosynthesis. Two QTL increasing Pn have been reported using progenies of Koshihikari × Habataki rice cultivars; one of these QTL on chromosome 4 also increases LNC^18^. Takai *et al*.^19^ reported that a partial loss-of-function by amino acid substitution in the *NARROW LEAF 1* (*NAL1*) gene, or a decreased NAL1 protein level due to the *indica* Takanari allele, contributed to increase LNC, leaf Rubisco contents, and Pn. *NAL1* encodes a protein that might be involved in polar auxin transport and is known to control vein patterning and leaf blade morphology^20^. In addition, *indica* Takanari *NAL1* allele genotypes have smaller leaf size compared to *japonica* Koshihikari allele genotypes^19^ and the *tropical japonica* Daringan *NAL1* allele increases total spikelet number, flag leaf width, and grain yield in *indica* IR64 genotypes^21^. Thus, *NAL1* is a key gene for yield-related traits in rice. While *indica* alleles of *NAL1* might be beneficial in increasing Pn, it is not clear if the partial loss of *NAL1* can increase whole-leaf photosynthesis (wPn; μmol CO_2_ leaf^−1^ s^−1^), as this allele might also reduce leaf area. In many plant species, leaf area is negatively correlated with Pn^22^ and, therefore, evaluation of wPn is essential for determining the photosynthetic ability of plants. Furthermore, in order to improve photosynthetic productivity, canopy photosynthesis also needs to be taken into consideration.

In this study, we aimed to detect novel QTL controlling Pn by increasing LNC. We screened chromosome segment substitution lines (CSSLs) developed from *japonica* Koshihikari and *indica* Nona Bokra rice cultivars (Takai *et al*., 2007), which harbour unique genes for salt tolerance, extremely late heading, and yield-related traits^23–26^. We used chromosome-substituted lines (SLs) containing the quantitative trait locus contributing the most to increase LNC to elucidate its effect on whole-leaf and canopy net photosynthesis.

## Results

### Detection of QTL increasing leaf N content in flag leaves and selection of SLs

**Figure 1:**
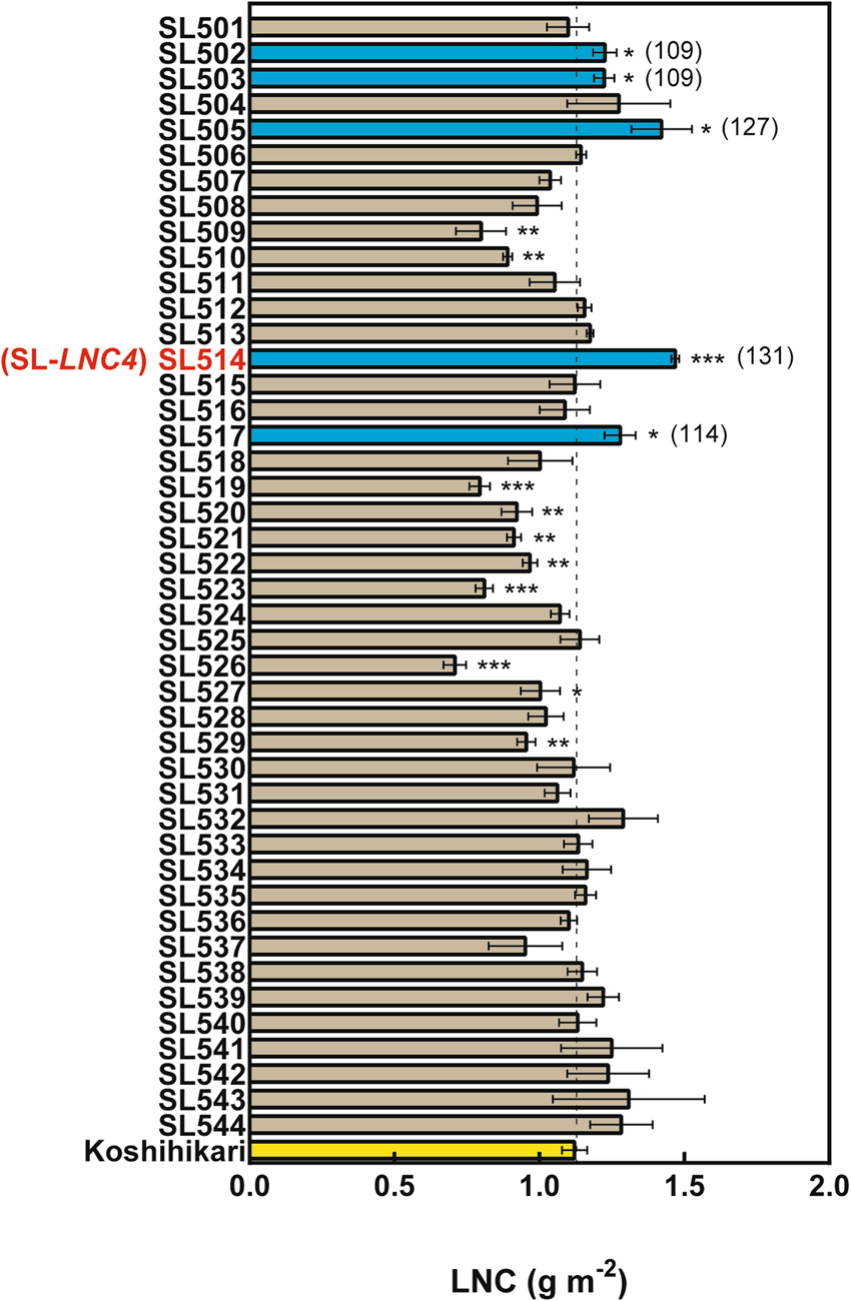
Leaf N content (LNC) in the flag leaves of Koshihikari/Nona Bokra CSSLs and the control accession Koshihikari. Columns indicate the mean ± standard deviation of LNC in three to four independent replicates. The yellow bar and the dotted line indicate the control. Blue bars indicate a significantly increase compared to control (*P* < 0.05) while grey bars indicate no significant increase. Values in parentheses indicate the percentage of the mean value of LNC in Koshihikari. *,**, and *** indicate significant differences in relation to Koshihikari at *P* < 0.05, 0.01, and 0.001, respectively.

Measuring the N content of flag leaves in CSSLs allowed identifying three QTL increasing LNC (*qLNC*) on chromosome (chr.) 1 (*qLNC1*), chr. 2 (*qLNC*2), and chr. 4 (*qLNC4*), and four QTL decreasing LNC on chromosomes 3, 6, 7, and 8 in Nona Bokra allele (Fig. 1 and Supplementary Fig. S1). Because SL514, which is the SL covering the *qLNC4* region in the Nona Bokra allele, demonstrated the greatest increase in N content (Fig. 1), we designated this SL as SL-*LNC4* in further analysis.

### Log of odds ratio (LOD) analysis and annotation of *qLNC4*

**Figure 2:**
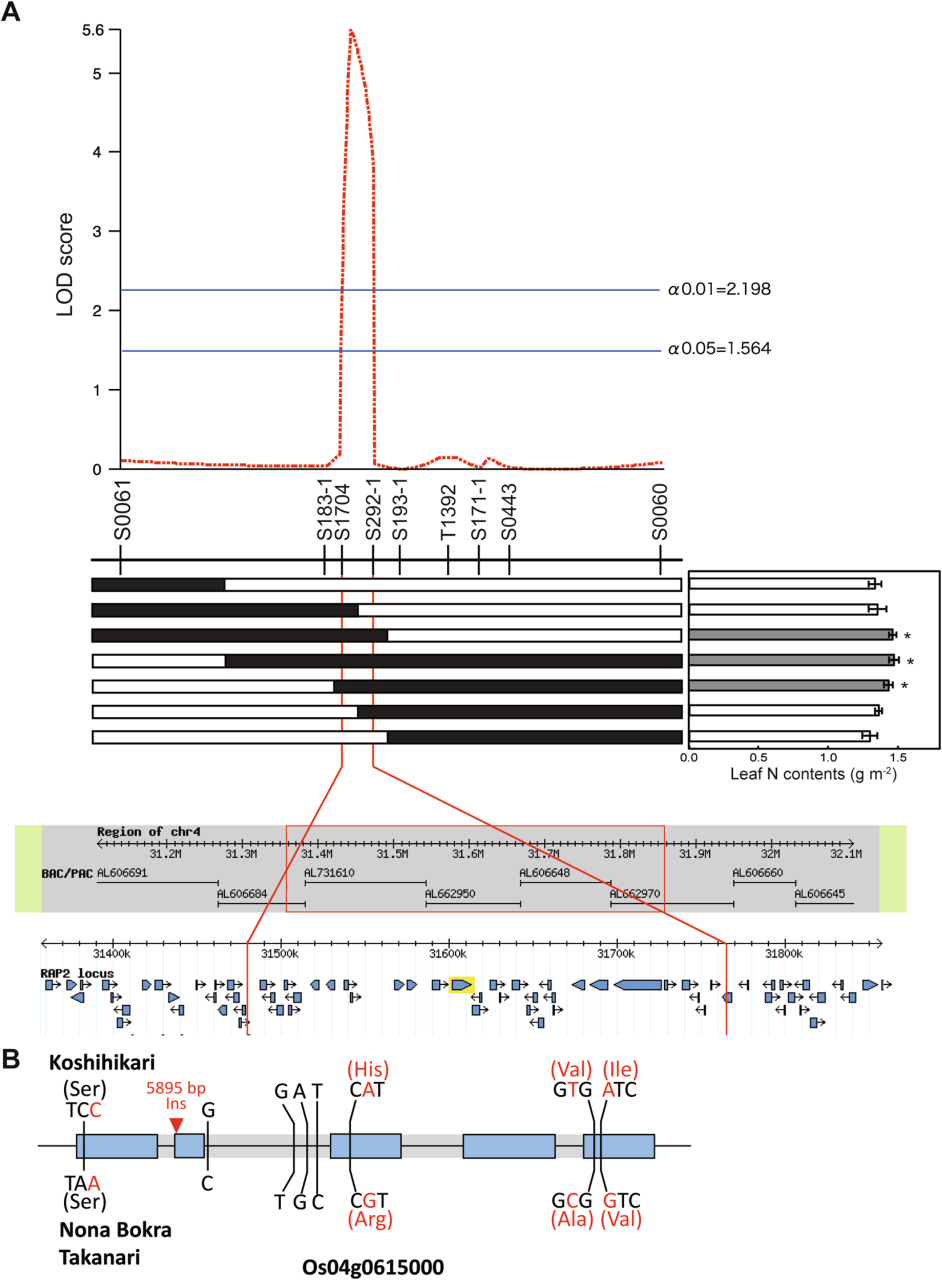
(**A**) Map-based cloning of *qLNC4*. Log-odds (LOD) scores were calculated in QGene 4.2.0 and gene annotations in the *qLNC4* region were obtained from RAP-DB. Filled and open bars represent homozygous chromosomal segments for Nona Bokra and Koshihikari, respectively. (**B**) Gene structure and variable sites of *NAL1* (Os04g0615000) in Koshihikari, Nona Bokra, and Takanari cultivars. Exons are indicated in light blue and nucleotide substitutions within exons are indicated in red. The 5,895-bp insertion in Koshihikari is also indicated in red.

LOD scores were calculated with a threshold of 2.198 (α = 0.01), and the *qLNC4* locus was restricted to a 286-kb region between markers S1704 and S292-1 (Fig. 2). This region is predicted to have 33 genes in the Rice Annotation Project Database (RAP-DB, http://rapdb.dna.affrc.go.jp/) (Supplementary Table S1)^27^, and *NAL1* is located within this region^20^. Four nucleotide substitutions were identified in the exon of *NAL1* in Koshihikari and Nona Bokra, and three of these changes are non-synonymous amino acid substitutions (Fig. 2). The Nona Bokra allele of *NAL1* was identical to the previously reported Takanari allele^19^.

### Photosynthetic characteristics and leaf N content in SL-*LNC4*

**Figure 3:**
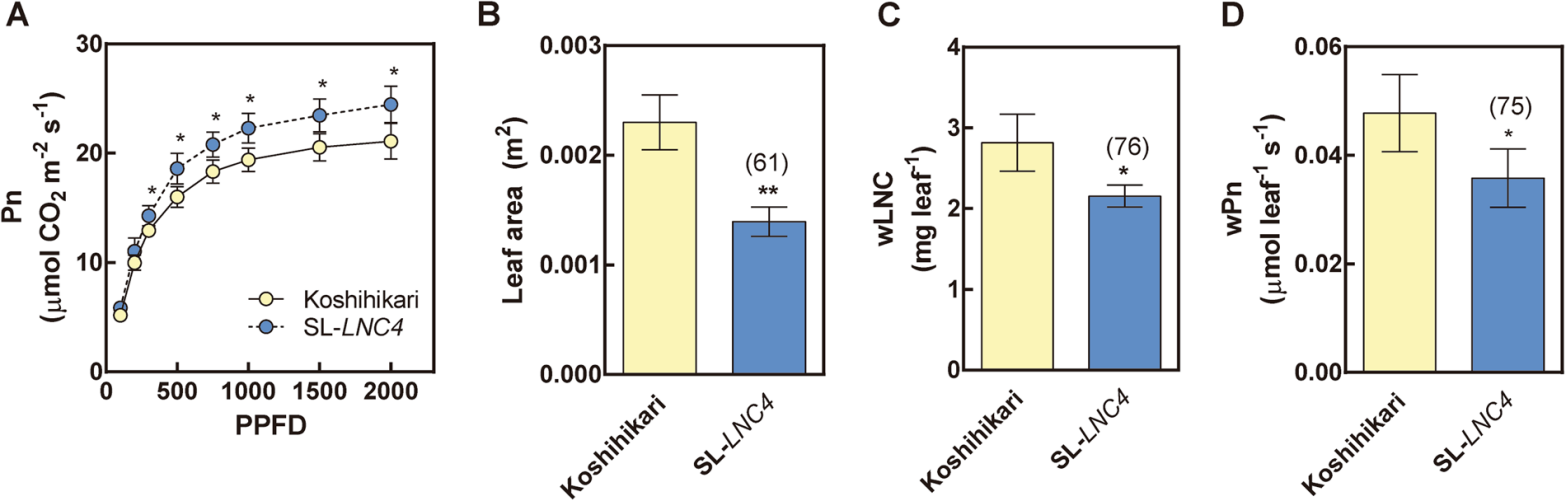
Light response of Pn (**A**), leaf area (**B**), wLNC (**C**), and wPn (**D**) of flag leaf blades in Koshihikari and SL*-LNC4*. Measurements were made in the field at a leaf temperature of 30 °C and external CO_2_ partial pressure of 37 Pa. The light response curve of Pn (**A**) was measured at PPFDs between 0 and 2,000 μmol m^−2^ s^−1^, and light saturated Pn (**B**,**C**,**D**) were measured at PPFD = 1,500 μmol m^−2^ s^−1^. Data are means ± standard deviations from three independent replicates. Yellow represents Koshihikari and blue represents SL*-LNC4*. wLNC was calculated using leaf area and LNC. wPn was calculated using leaf area and light saturated Pn. * and ** indicate significant differences in relation to Koshihikari at *P* < 0.05 and 0.01, respectively.

At heading, flag leaves of SL-*LNC4* had significantly higher Pn at various light intensity conditions compared to Koshihikari (Fig. 3A), but significantly smaller leaf areas (Fig. 3B). Although SL-*LNC4* had a higher LNC (Fig. 1) than Koshihikari, it showed lower leaf N content at the basis of individual whole leaves (wLNC; mg leaf^−1^) (Fig. 3C). Moreover, SL-*LNC4* did not show increased wPn (Fig. 3D).

### Correlation analysis between leaf areas and LNC

**Figure 4:**
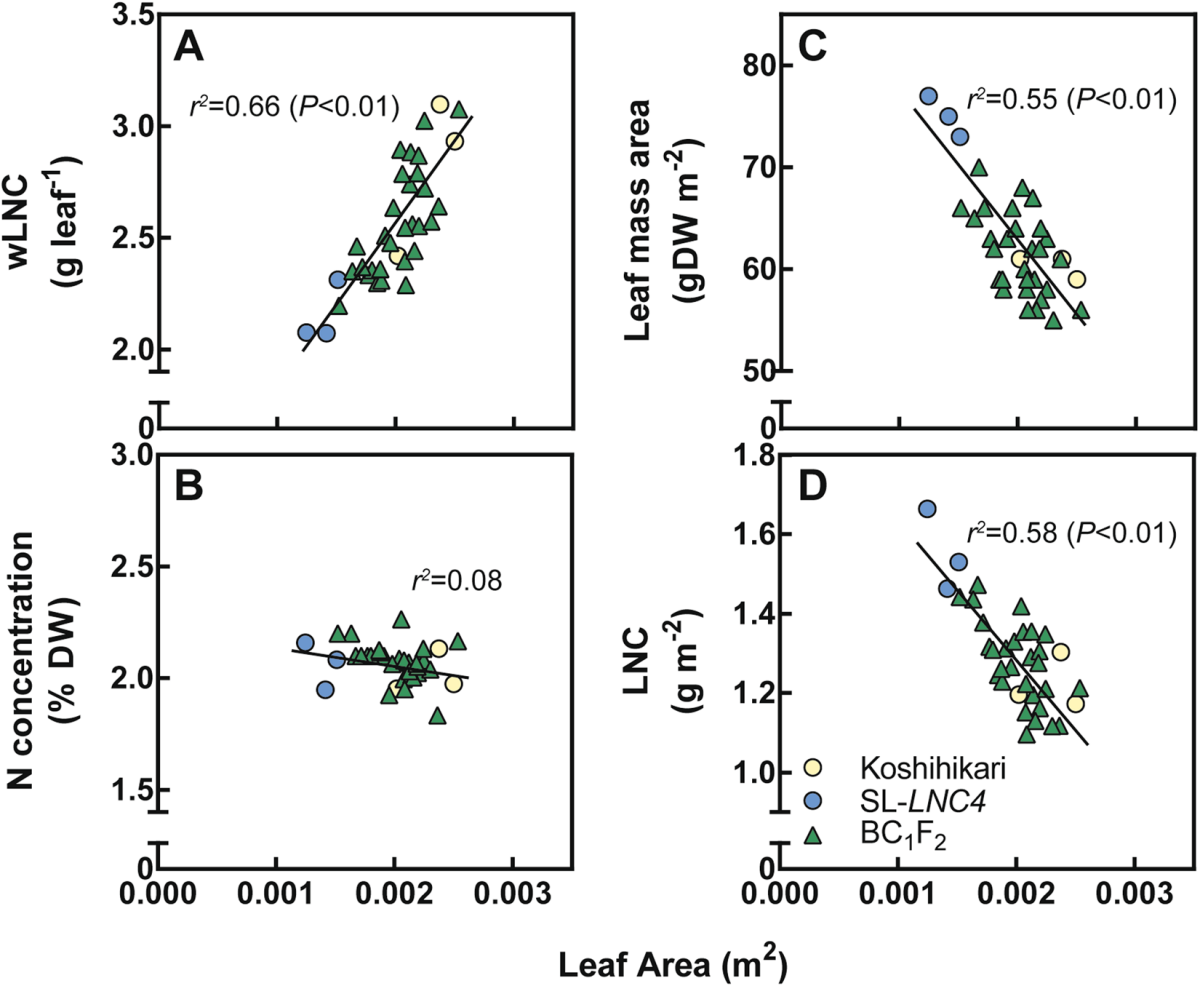
Relationship between the leaf area and wLNC (**A**), N concentration (**B**), leaf mass area (**C**), and LNC (**D**) of flag leaf blades. Yellow circles, blue circles, and green triangles represent Koshihikari, SL*-LNC4*, and the BC_1_F_2_ of Koshihikari × SL*-LNC4* crosses, respectively. For leaf area and wLNC, *Y* = 740*X* + 1.08, *r* ^2^ = 0.660. For leaf area and N concentration, *Y* = –81.0*X* + 2.23, *r* ^2^ = 0.080. For leaf area and leaf mass area, *Y* = –13200*X* + 88.8, *r* ^2^ = 0.549. For leaf area and LNC, *Y* = –324*X* + 1.94, *r* ^2^ = 0.581.

The correlations between leaf area and LNC, wLNC, and N concentration in flag leaves were analysed using 29 BC_1_F_2_ crosses between Koshihikari and SL-*LNC4* and their parents. Variance in leaf area ranged from 0.001247 to 0.002537 m^2^. A significant positive correlation (*r* ^2^ = 0.66, *P* < 0.01) between leaf area and wLNC was found (Fig. 4A), while there was no correlation between leaf area and N concentration (Fig. 4B). Leaf area and leaf mass area (*r* ^2^ = 0.55, *P* < 0.01) and LNC (*r* ^*2*^ = 0.58, *P* < 0.01) showed significant negative correlations (Fig. 4C,D).

### Characterisation of leaf canopy and leaf senescence

**Figure 5:**
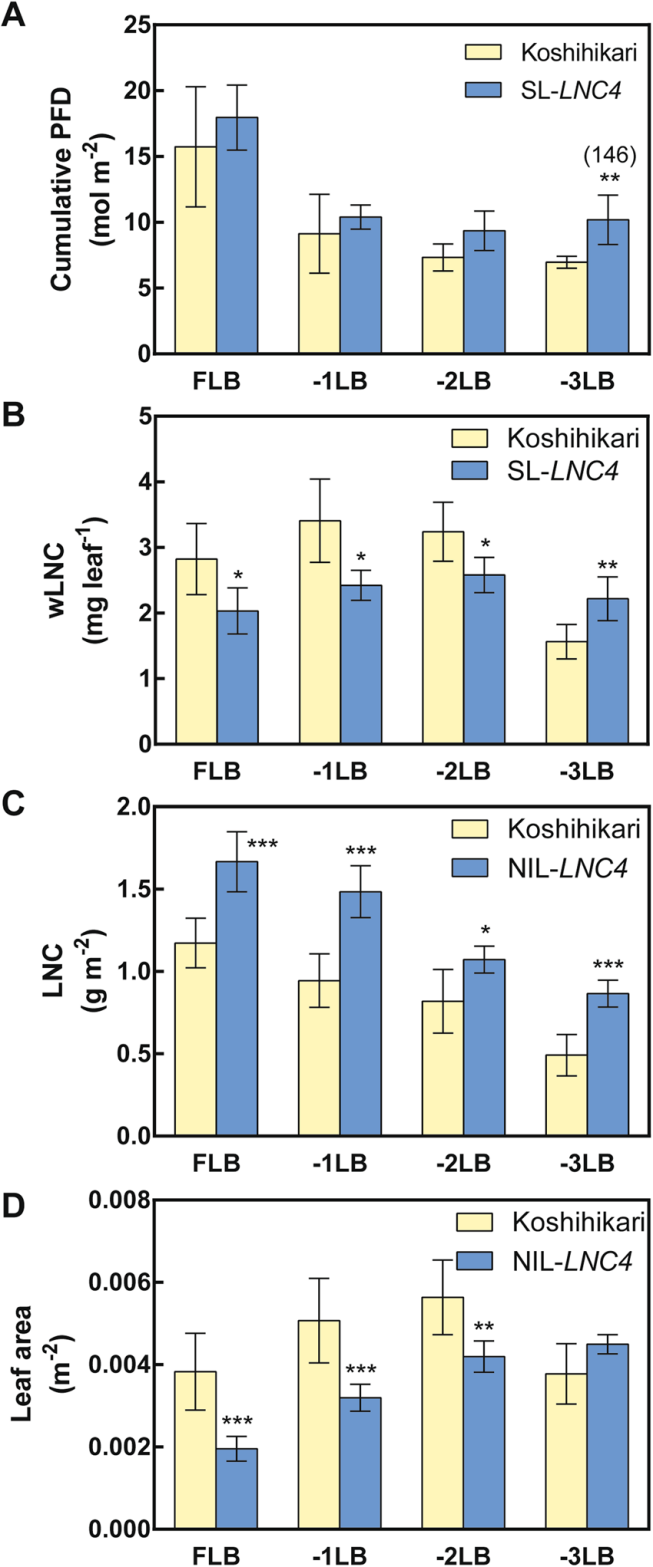
Cumulative PFD (**A**), wLNC (**B**), LNC (**C**), and leaf area (**D**) of leaves at different positions in Koshihikari and SL*-LNC4*. Yellow represents Koshihikari and blue represents SL*-LNC4*. FLB, −1LB, −2LB, and −3LB represent flag leaf blade, and first leaf blade, second leaf blade, and third leaf from the flag leaf downward, respectively. Data are means ± standard deviations of five independent replicates. *, **, and *** indicate significant differences in relation to Koshihikari at *P* < 0.05, 0.01, and 0.001, respectively.

**Figure 6:**
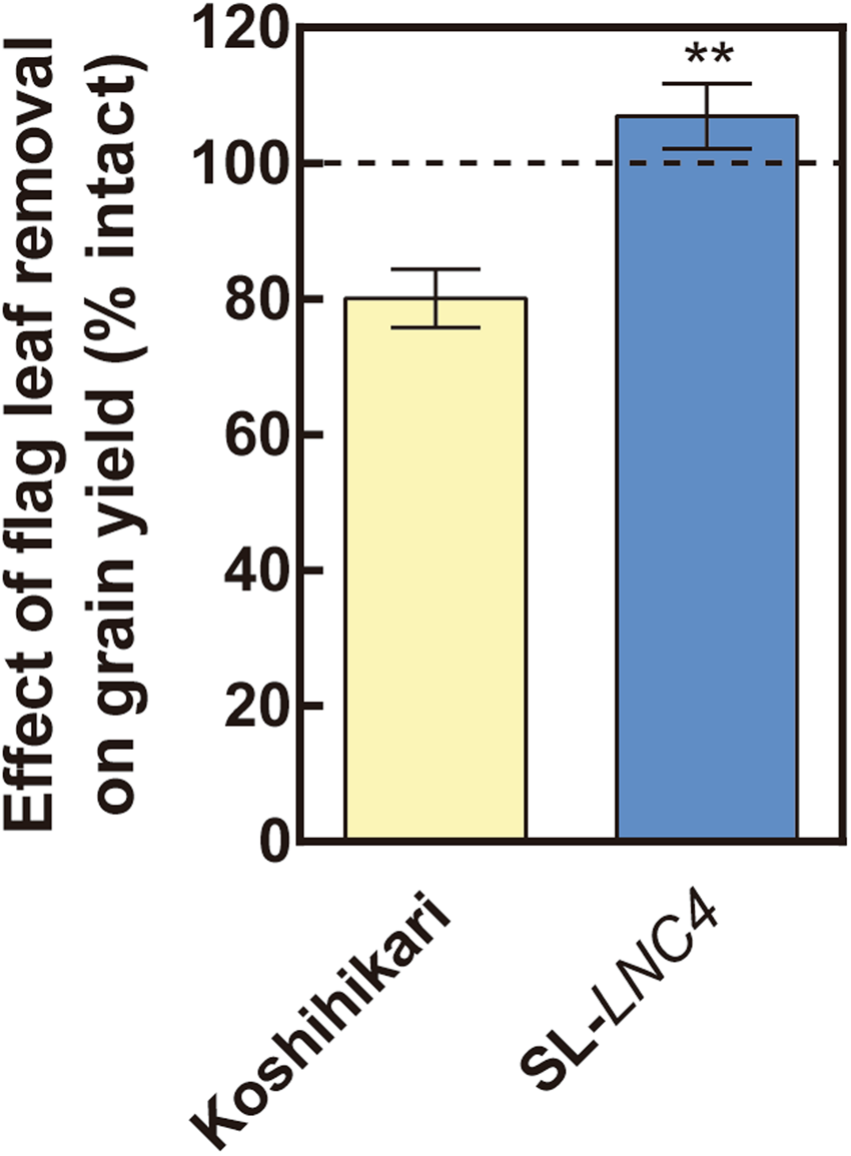
Effect of flag leaf removal on grain yield. All flag leaf blades were removed at heading, and grain yield per plant was compared to that of intact plants. Data are means ± standard deviations of five independent replicates. ** indicate significant differences in relation to Koshihikari at *P* < 0.01.

**Figure 7:**
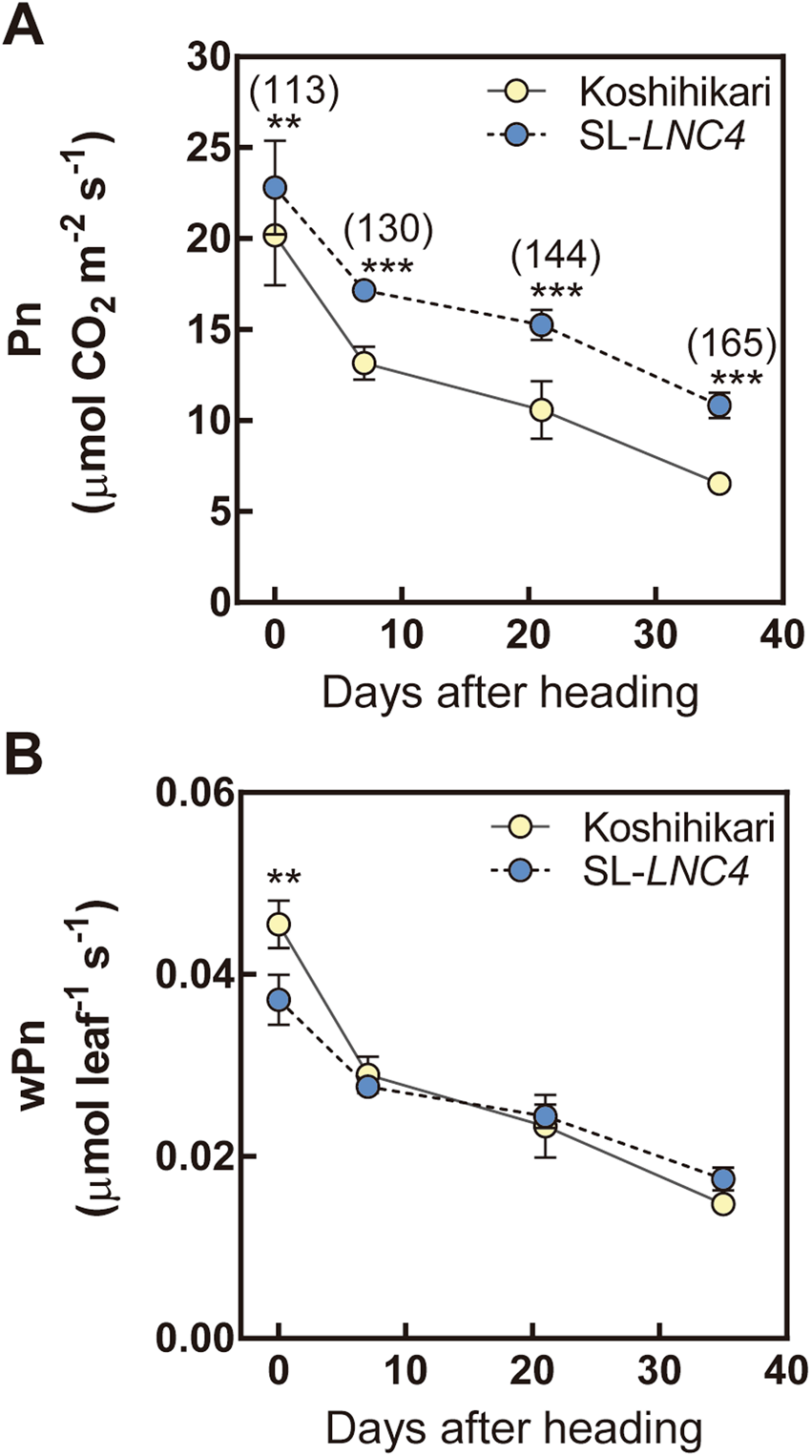
Changes in Pn (**A**) and wPn (**B**) in flag leaf blades of Koshihikari (control, yellow circle) and SL*-LNC4* (blue circle). Measurements were made in the field at a leaf temperature of 30 °C, PPDF of 1,500 μmol quanta m^–2^ s^–1^, and external CO_2_ partial pressure of 37 Pa. wPn were calculated using leaf area and Pn measured at the central portions of leaf blades. Data are means ± standard deviations of three to six independent replicates. Values in parentheses indicate percentages of the mean value of Koshihikari. ** and *** indicate significant differences in relation to Koshihikari at *P* < 0.01 and 0.001, respectively.

**Figure 8:**
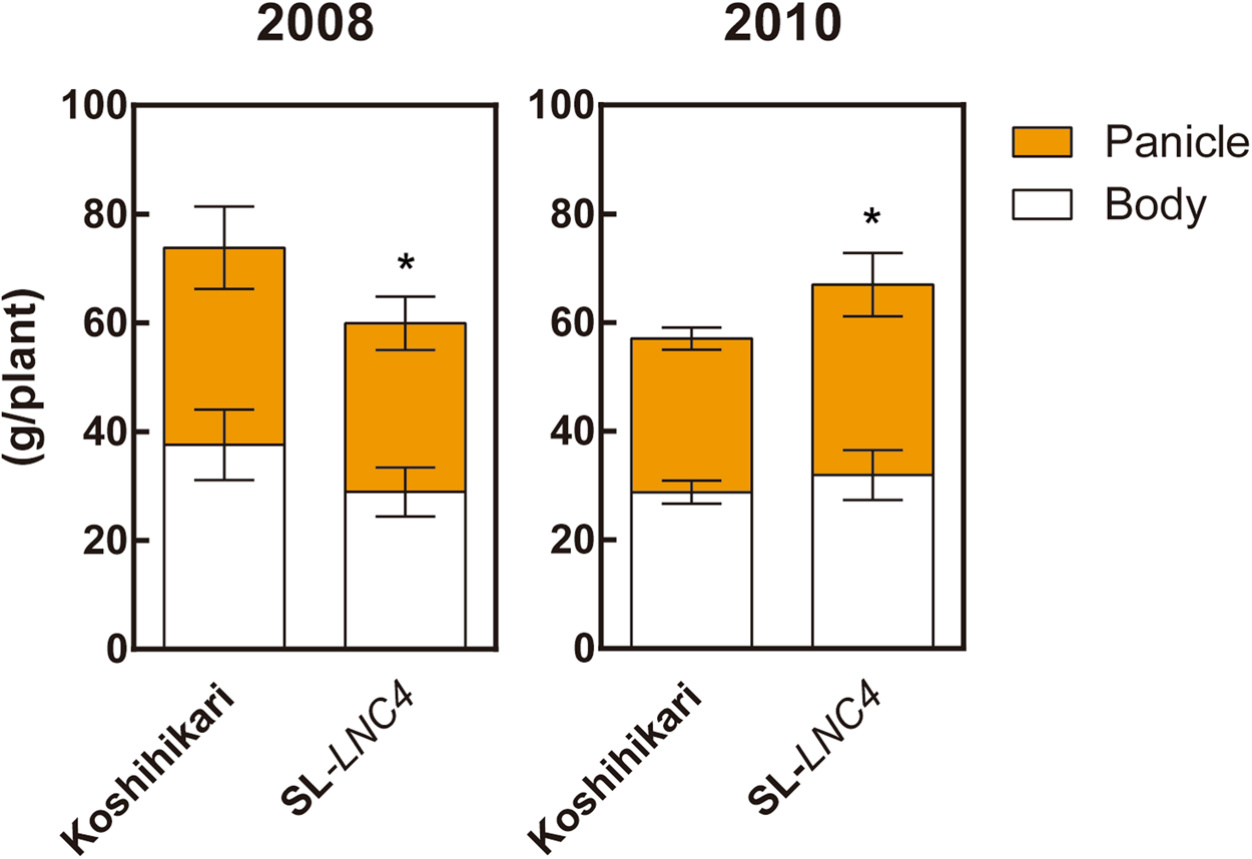
Biomass of Koshihikari and SL-*LNC4* in 2008 and 2010. Data are means ± standard deviations of five independent replicates. The biomass of the plant body is indicated in white and that of the panicle is indicated in orange.

The use of light sensitive films revealed that the third leaf blade from the flag leaf (−3LB) in SL*-LNC4* has higher photosynthetic photon flux density (PPFD) and intercepts more radiation at lower canopy layers than Koshihikari (Fig. 5A). SL*-LNC4* had significantly lower levels of wLNC from the FLB to the second leaf blade from the flag leaf (−2LB), while its −3LB had significant higher wLNC than Koshihikari (Fig. 5B). SL*-LNC4* had significantly higher levels of LNC than Koshihikari at all levels (Fig. 5C). Upon removal of flag leaves, Koshihikari demonstrated a decrease in grain yield of nearly 20%, while SL*-LNC4* was not affected (Fig. 6). Differences in Pn between SL*-LNC4* and Koshihikari increased with days after heading, from 113% at heading to 165% at 35 days after heading (Fig. 7A). Although SL*-LNC4* showed significant lower wPn at heading, there was no significant difference between Koshihikari and SL*-LNC4* after seven days post-heading (Fig. 7B). The biomass trial conducted at two different years showed that SL-*LNC4* biomass in 2008 decreased in relation to that of Koshihikari, while there was no significant difference between the two lines in 2010 (Fig. 8A). Morphological traits such as plant height, crown width, tiller number, and days to heading did not differ between Koshihikari and SL-*LNC4* (Supplementary Table S2).

## Discussion

In this study, we identified the QTL that increased LNC in the *indica* cultivar Nona Bokra allele (Fig. 1 and Supplementary Fig. S1). Among them, *qLNC4* had the strongest phenotypic effect on increased LNC (Fig. 1) and was responsible for increasing Pn (Fig. 3A). Thus, plants with high Pn can be screened using LNC as an index. Because gas exchange components are affected by environmental conditions and are difficult to evaluate precisely, phenotype screening, such as that performed based on LNC, is a more reliable index for determining photosynthetic rates.

The Nona Bokra allele provides evidence that *NAL1* is located within the *qLNC4* region (Supplementary Table S1) and possesses the same allele as the Takanari cultivar (Fig. 2). Furthermore, high LNC (Fig. 1) and high Pn (Fig. 3A) were observed in SL-*LNC4*. Although SL-*LNC4* might harbour additional substitutions in other genes, these phenotypes were similar to the partial loss-of-function substitution in the Takanari allele of *NAL1* ^19^. These results suggest that *NAL1* might be responsible for the observed effects of *qLNC4* on LNC and Pn.

Although increases in LNC and Pn by partial loss-of-function of *NAL1* were observed in the present study and in the study by Takai *et al*.^19^, the present study confirms for the first time that an increase in LNC does not cause substantial increases in wLNC and wPn (Fig. 3C,D). SL-*LNC4* has a small leaf area (Fig. 3B), which is consistent with the small leaf area of the near isogenic lines (NILs) that contain the Takanari allele of *NAL1* (Fig. 6a in Takai *et al*.) or mutant line (Table I in Qi *et al*.). Since *NAL1* is known to encode proteins involved in leaf morphology^19,20,28^, it is imperative to assess wPn in order to evaluate the potential effects of *NAL1* on leaf photosynthesis.

We hypothesised that the observed increases in LNC and Pn in SL*-LNC4* are associated with a decrease in leaf area, because leaf area is negatively correlated with photosynthesis in a broad array of plant species^22^. To examine this hypothesis, we analysed the correlation between leaf area and LNC and found a significant positive correlation between leaf area and wLNC (Fig. 4A), possibly related to N content being constant irrespective of leaf area (Fig. 4B) and to leaf area directly affecting wLNC. On the other hand, leaf mass area (LMA) and LNC were negatively correlated with leaf area (Fig. 4C,D), suggesting that smaller leaves might be thicker or denser than larger leaves, resulting in an increased LNC. SL*-LNC4* might have a N-assimilating capacity similar to that of Koshihikari because these accessions are genetically identical except for *qLNC4*. These findings suggest that increased LNC and Pn by partial loss-of-function of *NAL1* might be caused by increased LMA, which does not result in larger wLNC and wPn for a given N supply.

In general, uppermost fully expanded leaves have higher LNC than lower senescence leaves, and larger leaf areas maximize the photosynthetic N-use efficiency of the plant^29^. The *indica* cultivar IR64 amino acid sequence for *NAL1* is identical to that of Nona Bokra and Takanari cultivars, and this allele codes for smaller flag leaf area through a partial loss-of-function mutation. NILs containing functional *NAL1* alleles with an IR64 background have been shown to increase flag leaf area, and grain number and yield^21^. A larger flag leaf area might also contribute to produce more carbohydrates that fill enlarged sinks. To improve grain yield, it is important to enhance both sink size and source capacities^10^. *NAL1* has been reported to have pleiotropic effects on leaf anatomy, photosynthetic rate per leaf area, spikelet number, and grain yield^19–21,30,31^. To further determine how flag leaf area, LNC, and Pn interact under the genetic control of *NAL1*, the molecular mechanisms of this regulation should be investigated.

In contrast, a smaller flag leaf area might decrease the extinction coefficient and allow greater penetration of light to the lower leaves in addition to erect leaves^15,17^, thereby optimizing canopy photosynthesis. To compare the light environment inside the canopy, we measured the top-down cumulative photon flux density (PFD) of the leaves in each leaf level and demonstrated that SL*-LNC4* can capture more light at −3LB compared to Koshihikari (Fig. 5A). In addition, the leaf N distribution changed and SL*-LNC4* had higher wLNC at −3LB than Koshihikari (Fig. 5B). This could be caused by the lower demand of N from upper leaves because of limited N-sink due to a smaller leaf area. Because the removal of flag leaves did not affect grain yield in SL*-LNC4* (Fig. 6), this lineage seems to be less dependent on flag leaves for carbohydrate production during grain filling than Koshihikari, which showed a significant reduction of grain yield when flag leaves were removed. Thus, Koshihikari likely depends on flag leaves for carbohydrate accumulation in grains whereas the lower leaves of SL*-LNC4* likely have high photosynthetic ability due to spatial changes in light and N distribution.

In addition to spatial changes in the light environment and N distribution, changes in senescence rate were also observed (Fig. 7A,B). SL*-LNC4* showed lower senescence in flag leaves than Koshihikari and this difference increased during the 35 days after heading. However, the smaller wPn at heading in SL*-LNC4* caught up with that of Koshihikari after seven days, presumably due to its slower rate of senescence. Thus, the temporal changes in leaf photosynthesis in SL*-LNC4* also altered the canopy photosynthesis. In addition, at the heading stage, SL*-LNC4* maintained more leaf area than Koshihikari at −3LB, probably because the later has a higher senescence rate (Fig. 5D). These spatial and temporal changes in N distribution might be whole-plant compensation responses to the reduced leaf area, and the deeper light penetration and/or smaller N demand from upper leaves might delay the senescence of lower leaves. These pleiotropic effects might be the result of trait trade offs aiming to maximize the photosynthetic carbon gain under a given N supply.

Biomass production seems to reflect all photosynthetic activities occurring in the entire canopy during growth, and these are likely affected by the surrounding environmental factors. The effect of the partial loss-of-function allele on biomass at maturity differed between years (Fig. 8). Compared to the average values of the last 10 years, 2008 had significantly fewer sunlight hours while 2010 had more sunlight hours and higher global solar radiation (Supplementary Table S3). Thus, the partial loss-of-function allele might be beneficial for biomass production at higher solar radiation conditions. Haplotypes of single nucleotide polymorphisms (SNPs) for *NAL1* differed between *indica* and *japonica* subspecies in 950 cultivated rice lines in RiceHap3 (http://www.ncgr.ac.cn/RiceHap3). For example, the non-synonymous nucleotide substitution A8647 to G8647 (SNP ID: osc_rs528330) was found in 70.8% of *indica*, while 94.1% of *temperate japonica* rice had the A-allele. Natural variation of the *NAL1* gene in rice provides four major haplotypes, with *indica* characterized by the partial loss-of-function allele and *japonica* rice by the functional allele^32^. This suggests that both the functional *japonica* and the partial loss-of-function *indica* alleles might have beneficial effects on grain production at each favourable environment and might have been under positive selection during domestication. Thus, the benefit of plant types with either a (i) smaller flag leaf area coupled with higher Pn in lower leaves, or a (ii) larger flag leaf area with lower Pn in lower leaves, likely depends on factors such as N fertilization status, leaf orientation inside the canopy, and solar radiation at the cultivation latitude, among other environmental factors during the grain-filling period.

Results presented here suggest that plants’ strategy to improve overall canopy photosynthesis involves not only maximizing Pn in the uppermost fully expanded leaves, but also the spatial (lower leaves of the canopy) and temporal (senescence) control of Pn. Crop improvement and plant breeding should thus aim to maximize canopy photosynthesis, and *NAL1* is a possibly useful gene to target this trait.

## Methods

### Plant materials and detection of QTL increasing leaf N content

Seeds of 44 CSSLs with chromosomal segments of the Nona Bokra cultivar in the genetic background of Koshihikari^24^ and their parental lines were sown in May 2008. Fifteen seedlings of each line were transplanted in early June and grown under natural conditions in Tsukuba, Japan (latitude 36°N, longitude 140°E). To identify QTL for Pn, we first measured LNC of flag leaves. Using three to four plants per line, the flag leaves of three typical tillers were harvested from each plant just after heading, dried at 80 °C for 2 d, and weighed. Total N contents were measured with NC analyser (SUMIGRAPH NC-22F, Sumika Chemical Analyzer Service, Tokyo, Japan) as described in a previous study^25^. We chose CSSLs that showed a significantly (*P* < 0.05) higher or lower N content than Koshihikari and identified QTL from the graphical genotype data of these CSSLs^24^. We selected SL*-LNC4*, which carries a 15-Mbp chromosomal segment derived from Nona Bokra around *qLNC4* in the Koshihikari genetic background.

### Measurements of leaf N content and photosynthesis

CO_2_ assimilation rate was measured with a portable gas-exchange system (LI-6400, Li-Cor Inc., Lincoln, Nebraska, USA). The central portion of intact flag leaf blades of field-grown Koshihikari and SL*-LNC4* were used for measurements. They were obtained in a 2 × 3 cm chamber and between 11:00 and 13:00 in the field. Photosynthetic CO_2_ assimilation rates were measured when leaf temperature was 30 °C, reference CO_2_ concentration was 37 Pa, and relative humidity was 60%. The light response curve of leaf photosynthesis was obtained for PPFD values between 0 and 2,000 μmol m^−2^ s^−1^, and maximal CO_2_ assimilation rate was measured at PPFD = 1,500 μmol m^−2^ s^−1^ under a light emission diode (LED) source (red/blue 6400-02B LED source, Li-Cor Inc.). Flag leaf blades were sampled and dried at 80 °C after measuring leaf area (Automated Area Meter AAM-9, Hayashi, Tokyo, Japan). The N content of dried leaves was measured using the above-described method.

### Mapping and identification of the gene responsible for *qLNC4*

Using 768 BC_1_F_2_ plants resulting from SL*-LNC4* × Koshihikari crosses, we mapped *qLNC4* using markers designed by allele-specific primer polymerase chain reaction (PCR; Supplementary Table S4)^33^. Log-odds scores were calculated in QGene 4.2.0^34^ using a threshold based on 1,000 permutations at a 1% significance level. With homozygous recombinant plants, we delimited the *qLNC4* locus to a 286-kb region between markers S1704 and S292-1. Gene annotations for this region were obtained from RAP-DB^27^. This locus is predicted to have 33 genes, including *NAL1*, which is located within this region (Supplementary Table S1). The genomic sequences of both Koshihikari and Nona Bokra alleles of *NAL1* were sequenced in forward and reverse directions using the oligonucleotide primers listed in Supplementary Table S4.

### Correlation analysis between leaf area and LNC

To confirm the association between leaf area and N content of flag leaf blades, 29 BC_1_F_2_ recombinants of markers S0061 and S0060 were selected. At heading, the leaf area, dry weight, and N content of flag leaves from the main culm were measured according to the above-described methods, and correlations were analysed using BC_1_F_2_ plants and their parents.

### Analysis of light environment and canopy biomass

The integrated light level at the heading stage was measured using light-sensitive films (Opt leaf O-1D, Taisei Chemical Industries, Tokyo, Japan). The light transmittance of these films changes in proportion to the amount of accumulated light^35^ (Kawamura *et al*., 2005). Thirty-two Koshihikari and SL*-LNC4* plants were used for this experiment. Plants were distributed in four lanes of eight individuals each. Pieces of film were attached to the top flag leaf blade (FLB), first leaf blade below the flag leaf (−1LB), second leaf blade below the flag leaf (−2LB), and third leaf blade below the flag leaf (−3LB) of five plants within each lane. Light-sensitive films were not attached to plants in both ends of the lanes. Integrated solar radiation was intercepted on two consecutive days in August 2009, and light transmittance (*T*) was determined using a portable light meter (T-meter, Taisei Chemical Industries, Tokyo, Japan). The absorbance (*D*) of the film was calculated from *T* as follows:

1 $$D=a+b\{-{\rm{l}}{\rm{o}}{\rm{g}}\,(T/100)\}$$

where *a* and *b* are meter-specific constants (*a* = −0.0794, *b* = 1.2170). A film exposed to full light in an open area was used as a reference, and the cumulative photon flux density (PFD, mol m^−2^) was calculated through the calibration equation provided by manufacturer:

2 $${\rm{c}}{\rm{u}}{\rm{m}}{\rm{u}}{\rm{l}}{\rm{a}}{\rm{t}}{\rm{i}}{\rm{v}}{\rm{e}}\,{\rm{P}}{\rm{F}}{\rm{D}}=-0.4102\times (D/{D}_{0}\times 100)+45.11$$

where *D*_0_ and *D* are film absorbances before and after exposure, respectively.

Above-ground biomass at the heading stage was measured in 2008 and 2010. Plant height, crown width, tiller number, and days to heading were measured at the heading stage of the same plants in 2010. The daily average of sunlight hours (h) and average global solar radiation (MJ m^−2^) at Tsukuba in August 2008, 2010, and for the past 10 years (2001–2010) were obtained from Japan Meteorological Agency (http://www.jma.go.jp/jma/).

### Analysis of the effect of flag leaf removal

To examine the contribution of flag leaves to grain filling, all flag leaves were removed at heading from five plants of field-grown Koshihikari and SL*-LNC4* lines. After maturity, panicles were weighed, and the ratio of weight reduction of panicles to that of intact plants was calculated.

## Electronic supplementary material

Supplementary Information
