# Supplementary material for: Partial loss-of-function of NAL1 alters canopy photosynthesis by changing the contribution of upper and lower canopy leaves in rice
Source: Sci Rep. 2017 Nov 21;7:15958. doi: 10.1038/s41598-017-15886-5 (PMC5698313; doi:10.1038/s41598-017-15886-5)
Supplement: Supplementary file 1 — Supplementary Information [file 41598_2017_15886_MOESM1_ESM.pdf]

Supplementary Information

Partial loss-of-function of *NAL1* alters canopy photosynthesis by changing the contribution of upper and lower canopy leaves in rice.

Naoki Hirotsu[1,2], Kazuhiro Ujiie[2], Ishara Perera[1], Ayano Iri[1], Takayuki Kashiwagi[2], and Ken Ishimaru[2*]

[1] *Graduate School of Life Sciences, Toyo University, 1-1-1 Izumino, Itakura, Oura, Gunma 374-0193, Japan*

[2] *Institute of Crop Science, National Agriculture and Food Research Organization, Kannondai 2-1-2, Tsukuba, Ibaraki 305-8602, Japan*

[*] To whom correspondence should be addressed. Tel: +81 29 8388381. E-mail: kenshi@ affrc.go.jp

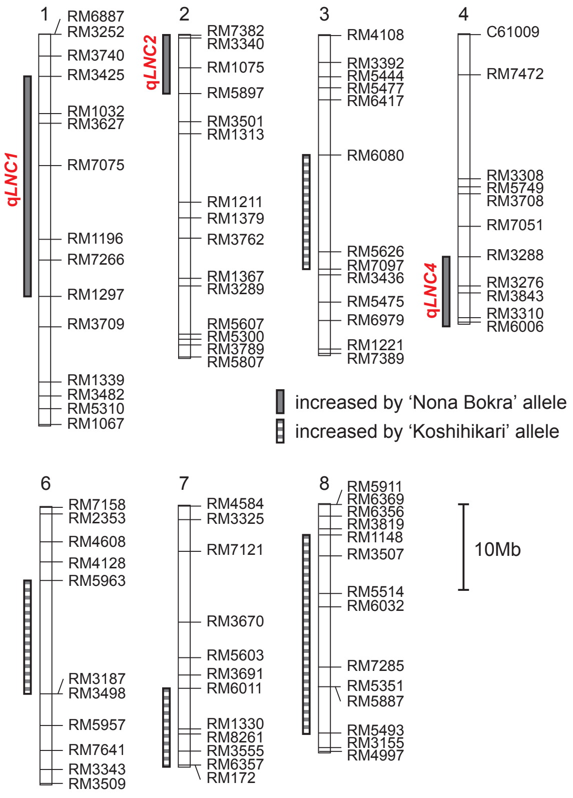

Supplementary Data Fig. S1 QTL map for LNC on the genetic map of Koshihikari/Nona Bokra CSSLs. Solid and broken lines with bars indicate positions of QTLs that increase LNC in the Nona Bokra allele and Koshihikari alleles, respectively. The threshold for the detection of QTLs is $P < 0.05$.

Supplementary Data Table S1 The list of putative loci within the QTL *qLNC4*. Annotated positions and descriptions were obtained by RAP2 (RAP-DB; http://rapdb.dna.affrc.go.jp/).

Name	Position	Description
Os04g0613200	chr4:31487802..31491236 (+ strand)	Virulence factor, pectin lyase fold family protein.
Os04g0613300	chr4:31494161..31496236 (− strand)	Conserved hypothetical protein.
Os04g0613400	chr4:31497453..31501264 (− strand)	Cell differentiation proteins, Rcd1−like family protein.
Os04g0613500	chr4:31501850..31503876 (+ strand)	Similar to 40S ribosomal protein S11.
Os04g0613600	chr4:31504527..31506463 (+ strand)	Ribosomal protein S17 family protein.
Os04g0613700	chr4:31507456..31512008 (− strand)	UTP−−glucose−1−phosphate uridylyltransferase family protein.
Os04g0613800	chr4:31517947..31522687 (− strand)	Methyl−CpG binding domain containing protein.
Os04g0613900	chr4:31526811..31531952 (− strand)	K+ potassium transporter family protein.
Os04g0614000	chr4:31537761..31540179 (+ strand)	Similar to Peroxisomal 2,4−dienoyl−CoA reductase (EC 1.3.1.34).
Os04g0614100	chr4:31541635..31542737 (+ strand)	Transcription factor, MADS−box domain containing protein.
Os04g0614500	chr4:31567549..31573266 (+ strand)	Aminotransferase class−III family protein.
Os04g0614600	chr4:31575185..31580988 (+ strand)	Similar to Viroid RNA−binding protein (Fragment).
Os04g0614700	chr4:31590244..31594833 (+ strand)	Conserved hypothetical protein.
Os04g0615000	chr4:31602061..31613277 (+ strand)	Hypothetical protein. (*NAL1*)
Os04g0615100	chr4:31613672..31618106 (+ strand)	Similar to Lecithine cholesterol acyltransferase−like protein.
Os04g0615200	chr4:31618395..31619434 (− strand)	Conserved hypothetical protein.
Os04g0615500	chr4:31624586..31628116 (+ strand)	Protein of unknown function DUF794, plant family protein.
Os04g0615600	chr4:31629976..31630593 (+ strand)	onserved hypothetical protein.
Os04g0615700	chr4:31637721..31641966 (+ strand)	Argonaute and Dicer protein, PAZ domain containing protein.
Os04g0615800	chr4:31646399..31648148 (+ strand)	Stem cell self−renewal protein Piwi domain containing protein.
Os04g0615900	chr4:31649974..31652576 (− strand)	FAR1 domain containing protein.
Os04g0616000	chr4:31653379..31656470 (− strand)	Conserved hypothetical protein.
Os04g0616100	chr4:31658631..31660977 (− strand)	Tetratricopeptide−like helical domain containing protein.
Os04g0616200	chr4:31661646..31663529 (− strand)	Protein kinase−like domain containing protein.
Os04g0616250	chr4:31662269..31662752 (+ strand)	Non−protein coding transcript, unclassifiable transcript.
Os04g0616300	chr4:31673021..31680893 (− strand)	Protein kinase domain containing protein.
Os04g0616400	chr4:31683826..31694512 (− strand)	Similar to Receptor−like serine/threonine kinase.
Os04g0616700	chr4:31698361..31726581 (− strand)	Protein kinase−like domain containing protein.
Os04g0616800	chr4:31728272..31729875 (+ strand)	Conserved hypothetical protein.
Os04g0616900	chr4:31738724..31743271 (+ strand)	Conserved hypothetical protein.
Os04g0617050	chr4:31748197..31748922 (− strand)	Auxin responsive SAUR protein family protein.
Os04g0617200	chr4:31752319..31752943 (− strand)	Conserved hypothetical protein.
Os04g0617400	chr4:31756001..31756545 (+ strand)	Conserved hypothetical protein.
Os04g0617600	chr4:31762595..31767992 (− strand)	Similar to Cdc48 cell division control protein 48, AAA family.

Supplementary Data Table S2 Morphological traits in Koshihikari and SL-*LNC4* at heading stage.

	Plant height (cm)	Crown width (cm)	Tiller number	Days to heading (days)
Koshihikari	111.2 ± 0.9	7.44 ± 0.66	13.8 ± 0.4	73
SL-*LNC4*	110.7 ± 0.7	7.46 ± 0.66	15.0 ± 0.7	73
	n.s.	n.s.	n.s.	n.s.

Supplementary Data Table S3 Daily average of Hours of sunlight and Global solar radiation at August of 2008, 2010 and 10-years (2001-2010) average. * and ** indicate significant differences compared to 10-years average at $P < 0.05$ and 0.01, respectively.

	Hours of sunlight (h)	Global solar radiation (MJ/m²)
Aug. 2008	4.3 ± 3.5 *	15.4 ± 5.9 $^{n.s.}$
Aug. 2010	7.5 ± 3.4 **	19.6 ± 4.2 **
10-years ave.	5.6 ± 1.5	16.9 ± 1.9

Supplementary Data Table S4 List of oligonucleotide primers used in this study.

Name	Forward primer (5'-3')		Reverse primer (5'-3')
SNP genotyping markers for map-based cloning of qLNC4. *Allele specific forward primers and common reverse primers are listed (see Hirotsu et al. 2010).*			
Marker	*'Koshihikari' allele specific primer*	*'Nona Bokra' allele specific primer*	*Reverse primer*
S0061	GTTCGTTTCATGCGGTGGA	GTTCGTTTCATGCGGTGGG	ACGGCTTTGATGGATCGAGA
S183-1	TGAGTACTCTAAGCTACTCCTATCATAGCTG	TGAGTACTCTAAGCTACTCCTATCATAGCTT	TGTGGTACCTTTGCTTGTGC
S1704	GTTTCACTCTGGTCACCTCCTTGAA	GTTTCACTCTGGTCACCTCCTTGAC	TTCCAGCTCACGTGGTTTCA
S292-1	CCTCTTCATCCAAGGTCG	CCTCTTCATCCAAGGTCA	GGCAAGTGATGCATAGAGCA
S193-1	TGAAGCATGGAAATACTCTTTGA	TGAAGCATGGAAATACTCTTTGG	ACCATTGACAGTGGCATTCA
T1392	CCATTTTCCATCTCCAAGTGTC	CCATTTTCCATCTCCAAGTGTT	GCTGCTCCTTTGTGGAACTC
S171-1	GAGTTGTGTGGGAATAGACCCC	GAGTTGTGTGGGAATAGACCCG	GCGCCTAGAACAATGGTAGC
S0443	CATTTTTATGGGGTGCCATCC	CATTTTTATGGGGTGCCATCA	CGCAGAATTCCTCAGCTTGAA
S0060	CACAACCAACATGGCTTCGCA	CACAACCAACATGGCTTCGCG	GGCAGGCTTTTCAGCATATC
Sequence of NAL1 gene			
NAL1-1	ACTCGACCGTCTGGAACTTG		CCAGCACTTTTTGCACTTCA
NAL1-2	ACATATATGCCGCCCCATT		GGGAAAAGAATCAACCTTGGA
NAL1-3	TGTCCAGATTTGCTGTGACC		TGCAATCCGGCATTAATATG
NAL1-4	TTCTTGAGGTGCGGAAACTT		TTGAACGGTCCCCTTACAAC
NAL1-5	TGGAAGCACATTTGCATGAT		CTTAGACCGTCCCAAACCAA
